# Supplementary material for: Empirical methods for controlling false positives and estimating confidence in ChIP-Seq peaks
Source: BMC Bioinformatics. 2008 Dec 5;9:523. doi: 10.1186/1471-2105-9-523 (PMC2628906; doi:10.1186/1471-2105-9-523)
Supplement: Additional File 1 — A variety of html documents from the USeq web site detailing the available applications, their best usage, output file type descriptions, command line menus, etc. [file 1471-2105-9-523-S1.zip › USeqUserGuides/JavaDoc.zip!/JavaDoc/util/gen/Bin.html]

Bin


|  |  |  |
| --- | --- | --- |
| **Overview** \| **Package** \| **Class** \| **Use** \| **Tree** \| **Deprecated** \| **Index** \| **Help** | |  |
| PREV CLASS   **NEXT CLASS** | **FRAMES**    **NO FRAMES**     **All Classes** |  |
| SUMMARY: NESTED \| FIELD \| CONSTR \| METHOD | DETAIL: FIELD \| CONSTR \| METHOD |  |


---


## util.gen Class Bin

```
java.lang.Object
  util.gen.Bin
```

---

``` public class Bin extends java.lang.Object ```

Info and methods about a particular bin, used by the Histogram class.

---

**Constructor Summary**

`Bin(double start,
double stop,
Histogram histogram)`


| **Method Summary** | |
| --- | --- |
| `boolean` | `contains(double value)`             Returns true if the value is >= bin.start and < bin.stop, thus stop is exclusive, start is inclusive. |
| `boolean` | `count(double value)`             If value is contains() returns true and increments the bin hit counter, otherwise returns false. |
| `boolean` | `count(double value, double score)`             If value is contains() returns true and increments the bin hit counter and total otherwise returns false. |
| `int` | `getHits()` |
| `java.lang.String` | `getLabel()` |
| `java.lang.Double` | `getMean()` |
| `java.lang.Double` | `getMedian()` |
| `double` | `getMiddle()` |
| `double` | `getStart()` |
| `double` | `getStop()` |
| `java.lang.String` | `toString()` |

**Methods inherited from class java.lang.Object**

`equals, getClass, hashCode, notify, notifyAll, wait, wait, wait`

**Constructor Detail**

### Bin

```
public Bin(double start,
           double stop,
           Histogram histogram)
```


**Method Detail**

### contains

```
public boolean contains(double value)
```

:   Returns true if the value is >= bin.start and < bin.stop, thus stop is exclusive, start is inclusive.

---


### count

```
public boolean count(double value)
```

:   If value is contains() returns true and increments the bin hit counter, otherwise returns false.

---


### count

```
public boolean count(double value,
                     double score)
```

:   If value is contains() returns true and increments the bin hit counter and total otherwise returns false.

---


### toString

```
public java.lang.String toString()
```

:   **Overrides:**: `toString` in class `java.lang.Object`

---


### getLabel

```
public java.lang.String getLabel()
```

---


### getHits

```
public int getHits()
```

---


### getStart

```
public double getStart()
```

---


### getStop

```
public double getStop()
```

---


### getMiddle

```
public double getMiddle()
```

---


### getMean

```
public java.lang.Double getMean()
```

---


### getMedian

```
public java.lang.Double getMedian()
```


---


|  |  |  |
| --- | --- | --- |
| **Overview** \| **Package** \| **Class** \| **Use** \| **Tree** \| **Deprecated** \| **Index** \| **Help** | |  |
| PREV CLASS   **NEXT CLASS** | **FRAMES**    **NO FRAMES**     **All Classes** |  |
| SUMMARY: NESTED \| FIELD \| CONSTR \| METHOD | DETAIL: FIELD \| CONSTR \| METHOD |  |


---
